# New Optical Voltage Sensor Based on Closed-Loop Pockels Cell and Sliding Mode Observer: Theory and Experiments

**DOI:** 10.3390/s25175319

**Published:** 2025-08-27

**Authors:** Luis Miguel Quispe-Valencia, Ricardo Tokio Higuti, Marcelo Carvalho M. Teixeira, Claudio Kitano

**Affiliations:** Electrical Engineering Department, School of Engineering, São Paulo State University (UNESP), Ilha Solteira 15385-007, SP, Brazil; ricardo.t.higuti@unesp.br (R.T.H.); marcelo.minhoto@unesp.br (M.C.M.T.); claudio.kitano@unesp.br (C.K.)

**Keywords:** optical high-voltage sensor, sliding modes control, polarimetric interferometer, metrology, phase measurement

## Abstract

The increasing power demand in substations and the advancement of smart-grid technology point to optical voltage sensors (OVSs) based on the Pockels effect as an attractive solution to replace traditional coil instrument transformers, due to their advantageous characteristics of lower cost and installation space, absence of explosion risks, as well as nonlinear effects such as magnetic hysteresis. Regarding the measurement, our OVS presents excellent linearity, 3 kHz bandwidth, and high input impedance. The primary contribution of this paper is to demonstrate, for the first time, the efficiency of a versatile nonlinear digital controller, based on sliding mode theory, for the optical phase demodulation of an OVS. A simple proportional-integral feedback control is employed to prevent signal fading and generate the two quadrature signals required by the observer, which includes the nonlinear digital controller. Experimental results, for 60 Hz sinusoidal voltages with amplitudes exceeding the half-wave voltage of the OVS, prove that peak-to-peak relative errors remain below 0.8%, while total harmonic distortion (THD) relative errors are under 1.5% when compared to a commercial high-voltage probe used as a reference. These results confirm compliance with Class 1.0 of the UNE-EN 60044-7 standard and show strong potential for applications in power quality measurements.

## 1. Introduction

Conventional instrument transformers, such as the potential transformers (PTs), have been used to convert high voltage into a smaller amplitude signal, but major issues—such as numerous faults in these oil-insulated PTs, concerns about the safety of operators and the electrical plant during faults, nonlinear response because of core saturation, ferroresonance, high cost, setup time and substation grounding requirements—have motivated researchers to find alternatives for voltage sensing [1,2,3]. Furthermore, since digital control and protection systems were introduced into electrical power systems, the ability to operate at low output voltage levels using transducers and the ability to mitigate the effects of electromagnetic interference have become important.

On the other hand, in the field of power quality analysis, measurement and parameter storage devices are typically used at low voltage levels. Therefore, optical sensors applied to power quality monitoring for different generation technologies (wind, solar, hydroelectric, thermal, nuclear) require high precision and a relatively wide bandwidth. Additionally, since grid quality control is performed periodically, the equipment must be easy to install and remove from the electrical distribution network, as monitoring periods can last up to one week. The sensor must also be cost-effective to justify its deployment at multiple nodes within the distribution grid [4]. Thus, these voltage transformers, when integrated into the measurement chain, must not compromise the quality of the parameters being measured [5].

For these reasons, optical voltage sensors (OVSs) combined with light transmission by means of optical fibers provide attractive features—high accuracy, wide dynamic range, high bandwidth (fast response to transients), good electrical insulation and no susceptibility to electromagnetic interference along the transmission link connecting the sensor to the substation, ability to remotely and safely control power systems (high isolation), reduced maintenance, compact size, reduced weight, and low cost—making OVSs suitable for applications in smart grids [6,7,8,9].

Despite the existence of several research papers published on modern alternative and competing approaches, such as FBG-PZT-based OVS [5,10,11], nowadays most sensors employed in utilities are essentially developed around electro-optical modulators, which, in turn, are based on the electro-optical (or Pockels) effect [6,12,13,14,15,16]. Basically, the Pockels effect refers to the changes in the optical properties (dielectric permittivity/refractive index) of certain noncentrosymmetric crystals, induced by external electric fields, modulating the polarization state of the transmitted light. The degree of polarization change depends on the intensity of the electric field associated with the voltage to be measured. Not coincidentally, the input-output (I/O) characteristic curve of the Pockels-effect-based OVS exhibits a great similarity to the I/O curve of a two-beam interferometer (such as Michelson and Mach–Zehnder interferometers). This is because a Pockels cell, positioned in the optical intensity modulator configuration and optically excited along the two eigen-axes of its birefringent crystal [17], can be classified as a “polarimetric interferometer”. Consequently, most of the optical phase interferometry demodulation techniques available in the literature can be advantageously adapted to work with Pockels-cell-based OVSs [18,19,20].

In Table 1, three sensors based on FBG-PZT technology were compared. This technology leverages the reflected portion of the spectrum when light reaches the Bragg grating. When an electric field is applied to a piezoelectric element, it stretches or compresses the fiber coupled to it, resulting in a variation in the Bragg wavelength. It can be seen from the studies that this approach exhibits good peak-to-peak error performance, but the tests were conducted using signals with low THD (approximately sinusoidal). Therefore, the concern regarding bandwidth for the purpose of testing short-duration signals was investigated up to 2.5 kHz.

Pockels cell can be mounted in transverse or longitudinal configuration, if the external electric field is applied in the transverse or longitudinal direction to the light propagation direction [17,21]. When applied to OVSs, they can employ different crystalline materials, mainly bismuth germanate (Bi_4_Ge_3_O_12_, BGO) [6,13,14] and lithium niobate (LN, LiNbO_3_) [12,15,16]. Recently, materials such as lithium tantalate (LiTaO_3_) [22], deuterated potassium dihydrogen phosphate (KD_2_PO_4_ or DKDP) [23], and the hybrid gallium arsenide + barium titanate (GaAs−BTO) [24], among others, have also been tested, showing promising results.

The primary measurement challenge of Pockels-cell-based OVSs stems from environmental disturbances [25], as occurs in all interferometers. In practical applications, issues such as temperature variations and/or air turbulences (changing the refractive index of the light propagation medium and/or causing expansion/contraction of the crystal), low-frequency mechanical vibrations (producing slight deviations when the optical devices are direction-sensitive or polarization-sensitive to light propagation), and the presence of natural birefringence (which is highly sensitive to temperature drifts) produce the signal fading phenomenon [26,27,28]. In addition, drawbacks such as non-homogeneity of the electric field distribution (fringing effect), insertion losses in the coupling of light from the fiber to the crystal, light scattering, and diffraction of the laser beam crossing the crystal, among others, degrade the photodetected signal [29]. In mathematical modeling, signal fading is a random variable that varies slowly in time, typically at frequencies below 20 Hz. The study of robust interferometric detection techniques, immune to fading, is one of the main reasons why the research area of OVSs has demanded research efforts nowadays.

It is also necessary to mention that the I/O relationship of most Pockels-cell-based OVSs is nonlinear, generating a highly distorted version of the input signal at the outputs. Fortunately, such a signal still preserves all the information about the signal of interest and can be detected by appropriate techniques. In case of low modulation depth, linearization techniques are sufficient; however, in case of deep-phase modulation, phase unwrapping methods are required [30,31,32].

It is worth noting that all these drawbacks can be overcome by using strategies in terms of optical hardware and/or modern signal processing techniques and computational resources currently available. In short, despite all the challenges, the Pockels-effect-based OVS is viable due to the numerous advantages mentioned at the beginning of this section, which are far superior to those of its electromagnetic version (the PT).

Nonetheless, most of the OVS topologies presented in the literature emphasize in optical hardware, unnecessarily increasing complexity, as well as choosing crystals with high electro-optical coefficients, in order to operate in low optical phase modulation depths, which are easier to handle. There are not many arrangements with OVSs operating under multi-fringes regime, in which phase unwrapping algorithms are required to demodulate the optical phase [33], and thus the maximum voltage measurable by the Pockels OVS is limited to the half-wave voltage $V_{\pi }$. In such cases, a conservative attitude is usually assumed, not paying attention to the large number of interferometry methods published in the literature (albeit applied to measurements of different physical parameters).

Among the numerous articles involving laser interferometry, there are sensors that employ closed-loop techniques (mainly applying classic PID control), but not frequently applied to OVSs based on the Pockels effect. Thus, for example, Zhang et al. (2010) [34] proposed the use of digital control in order to make the OVS insensitive to fluctuations in light intensity; make the dynamic range independent of the half-wave voltage of the Pockels crystal; and correct the influences of the alignment error of the sensing element on the measurement accuracy [34]. However, in the period between 2013 and 2017, there were several articles involving creative closed-loop OVSs, with the application of modern control techniques, for example, in the design of a robust control to guarantee that the closed-loop OVS is exponentially stable with an $H_{\infty }$ performance level, to eliminate the effects of the nonlinearity, time-delay, and noise in practical engineering [35]. Other works, involving tools such as prescribed $H_{\infty }$ performance, linear matrix inequality (LMI), and others, were dedicated to implementing novel closed-loop signal detection methods of the OVSs, techniques for suppressing non-reciprocal errors to improve the dynamic performance, optimize the high-frequency performance of OVSs, control algorithms to suppress the nonlinearity caused by the non-ideal parameters of OVSs, and so on [36,37,38]. It should be noted, however, that the optical circuits used in these proposals were somewhat complicated, using Faraday rotators, IO phase modulators (OPMs), and other specialized devices. All these papers were proposed for closed-loop OVS by the same group of authors and, after this period, works of this nature became less frequent in the literature.

Most works employed linear control theory to mitigate the effects of nonlinearities in OVSs. Recently, innovative proposals have tested the feasibility of using modern nonlinear control tools for interferometers, whose I/O characteristic is nonlinear in nature. The objective of this change of paradigm is to discover details still hidden behind the nonlinearity of the interferometry system and its multiple stability points. Among these proposals, applications involving control with variable structure and sliding modes (VS/SM) [39,40,41] and Takagi–Sugeno (TS) fuzzy nonlinear control [42] systems have been successful. More specifically, the VS/SM control technique under high-gain-bandwidth product operation regime, here simply called high-gain approach (HGA) [25], has generated excellent results [43].

In fact, the closed-loop interferometry technique proposed in [43], utilizing VS/SM nonlinear control and inspired by the classic article [44] (which used linear control), has solved most of the problems that made the HGA method uncompetitive with other detection techniques (as pointed out by [45]) in the past. Sliding mode control (SMC) offered robustness to disturbances and parameter variations, simplified control structure, and the ability to facilitate reduced-order system design. However, as in [44], reference [43] used a phase modulator (a PZT transducer) to compensate for problems such as signal fading and limited dynamic range for optical phase detection, which had the disadvantage of pre-calibration in nm/V units at the entire frequency operation bandwidth.

In 2022, Felão et al. [46] proposed another architecture, conceptually very different from the previous one, in which the interferometer operated in open loop (and therefore, with extremely simple optical hardware). However, a novel nonlinear VS/SM observer system was proposed, which, for all intents and purposes, made the association equivalent to a high-dynamic-range HGA closed-loop interferometer [43]. The phase modulator used to both compensate and demodulate the optical phase shifts was implemented by software, within the digital observer (consequently, requiring no pre-calibration by using a primary displacement standard).

All the papers discussed above, using VS/SM or TS fuzzy nonlinear control, have been applied to the area of micro- or nanometric vibrations in solids. This work combines an open-loop interferometer with a nonlinear control strategy, but will be tested, for the first time, on high-voltage measurement. In particular, it is known that VS/SM control faces technical challenges, including chattering, matched and unmatched uncertainties, and non-modeled dynamics. Therefore, such issues will also be addressed here.

The consequences of high levels of voltage and current harmonic distortion in the component elements of power systems and in electrical loads are broadly known [47,48,49], because these systems are becoming increasingly sensitive to such distortions. The identification of the presence of harmonic components, as well as their quantification, is an important requirement in the evaluation of the quality of electrical energy in distribution networks, class 13.8 kV. Contributions to the area of power quality, in particular, in measurements and analyses of harmonic distortion of high-voltage signals, will also be presented in this work.

In relation to the standards used for instrument transformers, nowadays the International Electrotechnical Commission (IEC) proposes the use of IEC 61869-3:2011 for inductive voltage transformers [50] and IEC 61869-5:2011 for capacitor voltage transformers [51]. Optic transducers were included in the group of electronic voltage transformers and published in IEC 60044-7:1999 [2]. This old standard should be replaced by IEC 61869-7, but the IEC online site [52] has not officially launched it. Some valid standards were adapted from IEC 60044-7:1999 [53], such as European norm UNE-EN 60044-7:2001 [54] and Chinese standard GB/T 20840.7-2007 [35,55]. Several papers up to 2023 [56,57,58,59] still use the old standard as a valid reference, so this work also considers the old standard and those derived from it as valid references.

Essentially, the main objective of this contribution is to demonstrate that it is possible to measure voltage and perform spectral analysis using the dual-crystal configuration proposed in [19], by replacing the differential cross multiplication method by a sliding mode observer, adapted from the VS/SM approach proposed by [46], as the demodulation procedure. In this way, to the best of our knowledge, the application of this type of SMC controller for optical high-voltage sensing is an original contribution.

The essence of the schemes and mathematical foundations from [19,46] are preserved. Accordingly, the main novelties of this work are: (1) the use of a nonlinear strategy—never before applied to voltage measurements—to demodulate the optical phase of interest in a polarimetric interferometer; (2) the new challenges that our optical voltage sensor must overcome to perform AC voltage measurements at 60 Hz with harmonic content; (3) the ability to carry out spectral analysis; and (4) compliance with current standards.

## 2. Materials and Methods

### 2.1. Optical Voltage Sensor

**Figure 1:**
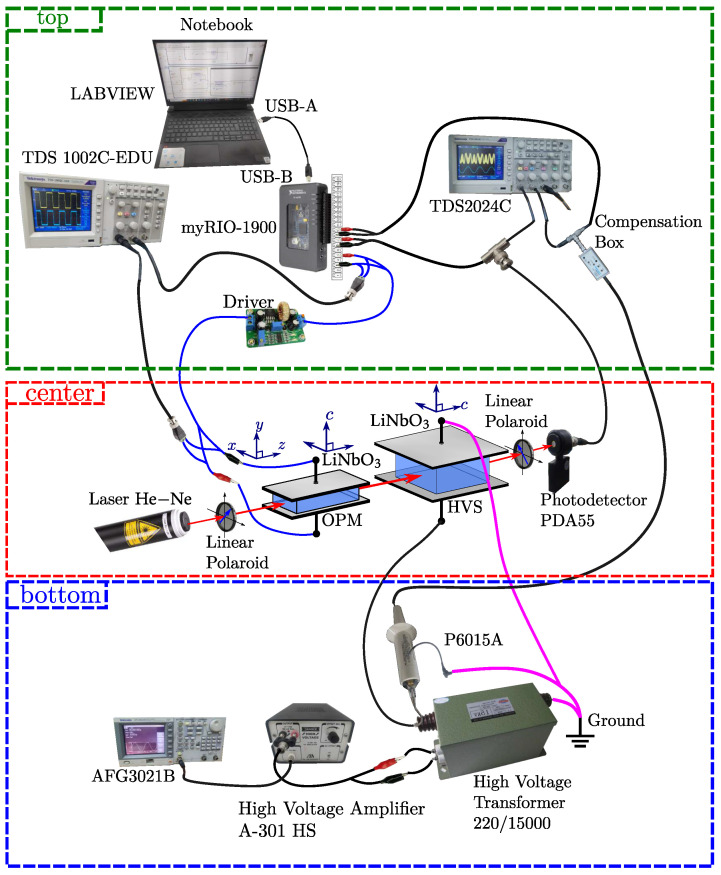
General schematic of the OVS and feedback control loop experimental setup: (**top**) signal processing system; (**center**) optical hardware; (**bottom**) high-voltage signal generation system.

The schematic diagram of the system is shown in Figure 1. The optical hardware (center) consists of a random polarized He−Ne laser (λ=632.8 nm, 20 mW, N-LHR-993, Newport, CA, USA); the cascaded association of Pockels cells based on LiNbO_3_ crystals (from Crystal Technology Inc., Palo Alto, CA, USA), positioned between two crossed dichroic polarizers; and a light photodetector (Thorlabs, PDA55, Newton, NJ, USA). One of these cells acts as a high-voltage sensor (HVS), and the other as an optical phase modulator (OPM). Figure 1 (bottom) illustrates the dry-type transformer that provides the high voltage to be measured, which has its primary winding connected to a digital signal generator (AFG3021B, Tektronix, Beaverton, OR, USA), in series with a pre-amplifier (A-301 HS, A. A. Lab Systems, Wilmington, DE, USA). A high-voltage probe (Tektronix P6015A, 28 kV, 75 MHz) connected to the HVS is used as a reference in the measurements. Figure 1 (top) illustrates the notebook used to implement the detection algorithms in LABVIEW (version 15.0, National Instruments) language and to program the embedded device (myRIO-1900, National Instruments, Austin, TX, USA) for acquiring and processing the photodetected signal, whose output feeds an amplifier Driver that supplies a feedback low voltage to the OPM. TDS1002C and TDS2024C oscilloscopes are used simply to assist in monitoring system adjustments and measurements.

LiNbO_3_ is a negative uniaxial crystal, whose ordinary and extraordinary refractive indices (measured in the optical range, at λ=632.8 nm) are $n_{o}=2.286$ and $n_{e}=2.200$, respectively [17]. The coordinate axes system (*x*, *y*, *z*) shown in Figure 1 (center) refers to the coordinate system of the interferometer, while (*a*, *b*, *c*) refers to the crystal axes, with *c* corresponding to the optical axis of LiNbO_3_. Both Pockels cells are in the transverse configuration; however, the vectors of the involved external electric fields are pointed in the directions of the *b* and *c* axes of the crystals of the HVS and OPM cells, respectively (or the *y*-axis of the interferometer), while the propagation directions of the optical beams are *c* and *a*, respectively (or the *z*-axis of the interferometer).

The OPM is a bulk LiNbO_3_ Pockels cell with dimensions of 10 mm, 1.1 mm, and 50 mm, in the *x*, *y*, and *z* directions, respectively. The HVS is also a bulk LiNbO_3_ Pockels cell, with dimensions of 20 mm, 9.92 mm, and 10.26 mm along *x*, *y*, and *z* directions, respectively. Following [60], the voltage that yields an optical phase retardation of π rad between ordinary and extraordinary modes is called the half-wave voltage. Thus, for the optic scheme of Figure 1, the theoretical half-wave voltage for OPM and HVS are $V_{\pi m}=64.92$ V and $V_{\pi S}=3.77$ kV respectively.

**Figure 2:**
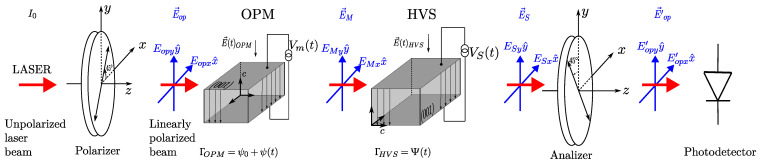
OVS composed of aligned Pockel HVS and OPM cells between crossed polarizers.

The propagation analysis of light through cascaded birefringent elements, such as the system shown in Figure 1 (center), can be treated systematically using the Jones matrix method [25,60]. In Figure 2, a full detailed diagram of the optical section corresponding to that in Figure 1 (center) can be seen, where the respective vectors of the external electric fields, $\vec{E}{\left(t\right)}_{OPM}$ and $\vec{E}{\left(t\right)}_{HVS}$, for OPM and HVS cells, as well as the respective voltages used to produce them, $V_{m}\left(t\right)$ and $V_{S}\left(t\right)$, can now be identified. In this configuration, the front polarizer is adjusted with its transmission axis at $45^{\circ }$ to the *x*-axis of the interferometer, in order to couple equal values of linearly polarized optical field to the sensor system along the fast and slow axes of both crystals. These beams are optically phase modulated by both cascaded cells; however, since their polarizations are perpendicular to each other, no interference occurs. For this reason, the rear polarizer (called the analyzer) is adjusted with its transmission axis at $-45^{\circ }$ from the *x*-axis, in order to promote interference between the polarized optical beams and, consequently, obtain information about the voltage to be measured, $V_{S}\left(t\right)$.

Historically, double-crystal OVS systems whose crystals, with similar characteristics (dimensions and material types) but with their respective optical axes (*z*-axis) in the opposition directions, were arranged in a cascaded configuration in order to cancel the effect of variation of the natural birefringence term and improve temperature stability [61,62,63]. Unlike most solutions presented in the aforementioned works, the objective of the OPM cell is not to cancel the effect of variation of the natural birefringence of the sensing crystal, but rather the effect of variation in its linear birefringence. Actually, since the sensing crystal was positioned in a transverse configuration with its optical axis aligned along the direction of light propagation, it does not present any natural birefringence term. So, by applying a periodic voltage to the OPM cell, with an amplitude controlled by the proportional-integral (PI) control system, aiming to generate two quadrature signals through synchronized acquisition, the OPM is able to compensate both the variations in the linear birefringence of the HVS cell and its own natural birefringence, suppressing the signal fading of the OVS. The advantage of this OPM is it being a bulk device (cheap and easy to manufacture) that operates with relatively small voltage values.

After the unpolarized incident beam passes through the front polarizer, the electric field vector of the laser beam (${\vec{E}}_{op}$) can be represented by the following Jones vector [60]:

(1) $${\vec{E}}_{op}=\left[\begin{array}{c} E_{opx}\\ E_{opy} \end{array}\right]=\frac{\sqrt{I_{0}}}{2}\left[\begin{array}{c} 1\\ 1 \end{array}\right],$$

where the optical intensity of the incident beam is assumed to be $I_{0}$ (W/m^2^). Since the transmission axis of the polarizer is at $45^{\circ }$ with respect to the *x*-axis, the electric field components along the *x*- and *y*-axes present the same amplitudes, i.e., $E_{opx}=E_{opy}$.

The ray emerging from the polarizer strikes a plane-parallel OPM cell. Upon entering the Pockels cell, the light is coupled to two crystal eigenmodes with different phase velocities of propagation, with their displacement vectors (C/m^2^) vibrating along crystalline *x*- and *y*-axes (which are mutually orthogonal) of the LiNbO_3_. As a result of this propagation, these rays emerge from the cell with a certain phase shift (or retardation) that we define as $\Gamma_{OPM}$. According to [60], the Jones matrix representation for birefringent bulk crystals can be expressed as a function of this phase difference as

(2) $$\left[\begin{array}{cc} e^{-i\frac{\Gamma_{OPM}}{2}} & 0\\ 0 & e^{i\frac{\Gamma_{OPM}}{2}} \end{array}\right].$$

The polarization state (in the principal axes *x*, *y*) of the emerging beam light at the output plane in the OPM cell is calculated by multiplying (1) by (2), as given by

(3) $${\vec{E}}_{M}=\left[\begin{array}{c} E_{Mx}\\ E_{My} \end{array}\right]=\frac{\sqrt{I_{0}}}{2}\left[\begin{array}{c} e^{-i\frac{\Gamma_{OPM}}{2}}\\ e^{i\frac{\Gamma_{OPM}}{2}} \end{array}\right].$$

Following the same procedure used for the OPM cell, rays linearly polarized along the *x*- and *y*-axes will acquire a phase retardation due to propagation through the Pockels HVS cell, defined by $\Gamma_{HVS}$. The Jones matrix representation of this birefringent element is then presented by

(4) $$\left[\begin{array}{cc} e^{-i\frac{\Gamma_{HVS}}{2}} & 0\\ 0 & e^{i\frac{\Gamma_{HVS}}{2}} \end{array}\right].$$

By multiplying (3) by (4), the output electric field of the HVS cell can be estimated as

(5) $${\vec{E}}_{S}=\left[\begin{array}{c} E_{Sx}\\ E_{Sy} \end{array}\right]=\frac{\sqrt{I_{0}}}{2}\left[\begin{array}{c} e^{-i\frac{\Gamma_{OPM}+\Gamma_{HVS}}{2}}\\ e^{i\frac{\Gamma_{OPM}+\Gamma_{HVS}}{2}} \end{array}\right],$$

where we can see that the contribution of the phase difference of each cell only needs to be added in the argument of the exponential function, highlighting the usefulness of the Jones representation in optical systems with multiple cascaded birefringent elements.

The analyzer adjusted with its transmission axis at $-45^{\circ }$ from the *x*-axis can be depicted by the Jones matrix as [60]:

(6) $$\left[\begin{array}{cc} 1/2 & -1/2\\ -1/2 & 1/2 \end{array}\right].$$

Then, the electric field transmitted to the photodetector can be computed by multiplying (5) by (6), obtaining

(7) $$\begin{array}{cc} {\vec{E^{\prime }}}_{op} & =\left[\begin{array}{c} E_{opx}^{\prime }\\ E_{opy}^{\prime } \end{array}\right]=\frac{\sqrt{I_{0}}}{2}i\sin\left(\frac{\Gamma_{HVS}+\Gamma_{OPM}}{2}\right)\left[\begin{array}{c} -1\\ 1 \end{array}\right] \end{array}$$

As ${\vec{E^{\prime }}}_{op}$ is the optical beam incident on the active area of the photodetector located after the analyzer, its optical intensity (W/m^2^) is calculated as ${\vec{E^{\prime }}}_{op}^{†}.{\vec{E^{\prime }}}_{op}$, where the dagger indicates the Hermitian conjugate. Using the result obtained in (7), the intensity can be written as follows:

(8) $${\vec{E^{\prime }}}_{op}^{†}.{\vec{E^{\prime }}}_{op}=\frac{I_{0}}{4}\left(1-\cos\left(\Gamma_{HVS}+\Gamma_{OPM}\right)\right).$$

The silicon detector PDA55 (photodetector used for detection of light signals) performs the conversion from optical to electrical energy, where the optical phase shifts $\Gamma_{HVS}$ and $\Gamma_{OPM}$ from the optical setup are converted into equivalent time-varying electrical phases in the detected signal. This intensity-to-electrical conversion provides the following generic voltage signal:

(9) $$v_{pd}\left(t\right)=M\left\{1-V\cos\left(\Psi \left(t\right)+\psi_{0}+\psi \left(t\right)+\vartheta \left(t\right)\right)\right\},$$

where *M* is a bias voltage taking into account the photodiode responsivity and the gain of the transimpedance amplifying circuit, and *V* is the fringe visibility (0<V<1, dimensionless), incorporated ad hoc to take into account any misalignment between laser polarization and/or beam propagation direction relative to the crystal axes. Ψ(t) is the phase shift induced by voltage to be measured in HVS, and ϑ(t) corresponds to the slow random variation of phase in time, caused by thermal environmental drifts, which disturb the ordinary and extraordinary refractive indices of the crystal. ψ(t) is the phase shift produced by analog output from the myRIO card (which is then amplified by the “driver” circuit) and fed back to the OPM cell, while $\psi_{0}$ is the corresponding random quasi-static phase shift induced by the natural birefringence of the OPM crystal.

In principle, the determination of voltage $V_{S}\left(t\right)$ can be obtained from Ψ(t) by solving the arc-cosine in Equation (9). However, as this task is not so simple, due to phase ambiguity problems (and also because it involves random variations of other phases producing signal fading), another strategy will be adopted. Following the strategy used in [19], the voltage $V_{m}\left(t\right)=V_{m}\cos\left(2\pi f_{0}t\right)$ is applied to OPM, where $f_{0}$ is the frequency, and the amplitude is chosen as $V_{m}=V_{\pi m}/4$. Consequently, the optical phase shift induced by the OPM can be written as [60]:

(10) $$\psi \left(t\right)=\pi \frac{V_{m}\left(t\right)}{V_{\pi m}}=\frac{\pi }{4}\cos\left(2\pi f_{0}t\right).$$

Similarly, the phase shift induced by the measured voltage $V_{S}\left(t\right)$ applied to the HVS is

(11) $$\Psi \left(t\right)=\pi \frac{V_{S}\left(t\right)}{V_{\pi S}}.$$

By substituting (10) in (9), defining

(12) $$\theta \left(t\right)=\Psi \left(t\right)+\psi_{0}+\vartheta \left(t\right)+\frac{\pi }{4},$$

and then subtracting *M* from $v_{pd}\left(t\right)$, the resulting signal becomes

(13) $$v\left(t\right)=-MV\cos\left(\frac{\pi }{4}\cos\left(2\pi f_{0}t\right)+\theta \left(t\right)-\frac{\pi }{4}\right),$$

where the calculation of *M* is explained in Appendix A. If $v_{pd}\left(t\right)$ is sampled with a period $T_{s}$, v(t) can be expressed as the discrete sampled signal:

(14) $$v\left(nT_{s}\right)=-MV\cos\left(\frac{\pi }{4}\cos\left(2\pi f_{0}nT_{s}\right)+\theta \left(nT_{s}\right)-\frac{\pi }{4}\right).$$

On the other hand, if $T_{s}$ is adopted as the half period of $V_{m}\left(t\right)$, we obtain

(15) $$T_{s}=\frac{1}{2f_{0}},$$

and so, by inserting (15) into (14), the expression simplifies to

(16) $$\begin{array}{cc} v(nT_{s}) & =\left\{\begin{array}{cc} -MV\cos\left(\theta \left(nT_{s}\right)\right) & \mathrm{If}\;n\;\mathrm{is}\;\mathrm{even}\\ -MV\sin\left(\theta \left(nT_{s}\right)\right) & \mathrm{If}\;n\;\mathrm{is}\;\mathrm{odd}. \end{array}\right. \end{array}$$

**Figure 3:**
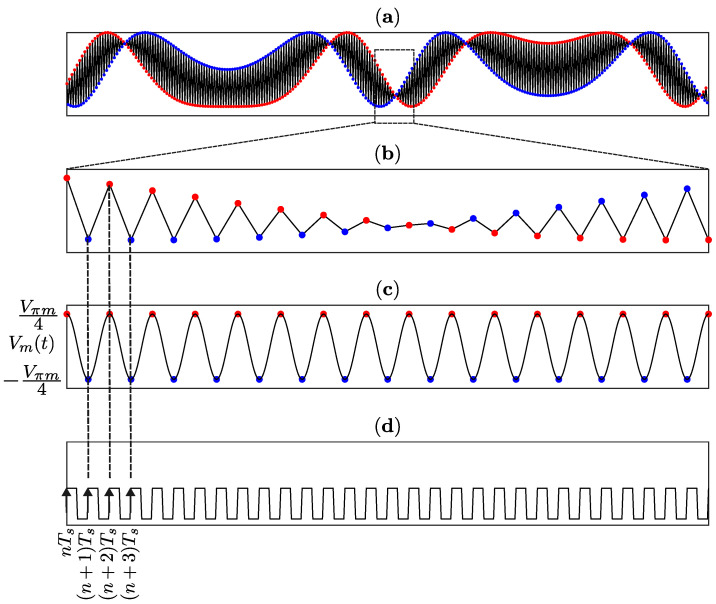
In-phase and quadrature envelopes of the interferometric signal and the interlaced acquisition: (**a**) photodetected signal $v_{pd}\left(t\right)$; (**b**) zoom photodetected signal $v_{pd}\left(t\right)$; (**c**) $V_{m}\left(t\right)$ voltage applied to OPM cell; (**d**) clock signal.

An important feature of the method is that the acquisition is synchronized with $V_{m}\left(t\right)$. In Figure 3a, in black color, a typical interferometric signal of the type described in Equation (14) [19] is illustrated. Although this signal is continuous in time, produced by a single photodiode, two distinct envelopes can be observed, in red and blue colors, if alternate acquisitions are considered. This observation is in accordance with (16), where red dots represent even sequence, and blue dots odd sequence, as shown in zoomed Figure 3b (where the sampling points were joined by straight-line segments to provide a better visualization of the two envelopes). Figure 3c shows that $V_{m}\left(t\right)$ has a period of $2T_{s}$, which is twice the sampling period, illustrated in Figure 3d, the clock signal. Notably, the even sequence is acquired when $V_{m}\left(t\right)=V_{\pi m}/4$ (corresponding to the crests), while the odd sequence reaches the lowest value $V_{m}\left(t\right)=-V_{\pi m}/4$ (the troughs). Unlike other classic methods, which use two photodiodes [64,65], this interlaced acquisition allows one to obtain the two envelopes from a single photodetector, and, since phase difference is π/2 rad, they are orthogonal with each other, that is, they are in phase quadrature.

**Figure 4:**
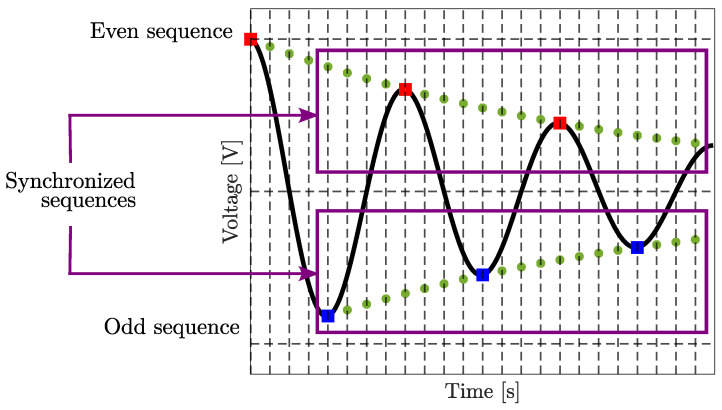
Interpolation and synchronization of even and odd sequences.

The signals in (16) are not acquired simultaneously; instead, there is a $T_{s}$ delay between the even and odd sequences. To address this, the manipulated signal must be corrected through interpolation and time-shifting. The interpolation process inserts ($L_{e}-1$) additional points (green dots in Figure 4) between each pair of original consecutive square dots (of the same color). By applying a $L_{e}/2$-point delay to the even sequence, the two sequences become synchronized, with a new effective sampling rate of $L_{e}/2$ times the original sampling frequency. Figure 4 illustrates this process: the red and blue square dots represent the raw data captured by the myRIO, while the green circular dots denote the interpolated points. For this study, $L_{e}=8$ and $f_{0}=15$ kHz, resulting in a synchronized sampling rate of 120 kHz for both sequences.

After normalizing the signals in (16), we have:

(17) $$\begin{array}{cc} p_{1}\left[n\right] & =-\cos\left(\theta \left(nT_{s}^{\prime }\right)\right)\\ p_{2}\left[n\right] & =-\sin\left(\theta \left(nT_{s}^{\prime }\right)\right), \end{array}$$

where $T_{s}^{\prime }$ is the new sampling period after interpolation. The normalizing procedure is performed by dividing (16) by MV, which is computed by the Mean(∗) block (explained in Appendix A).

**Figure 5:**
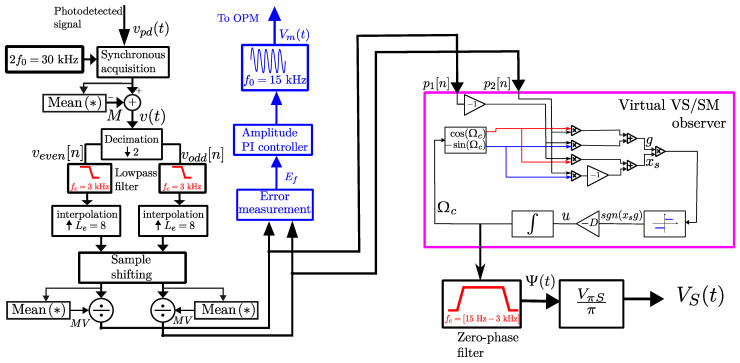
Schematic diagram of the proposed method, feedback control system, and VS/SM nonlinear observer.

In Figure 5 (left, in black), the procedure for obtaining $p_{1}\left[n\right]$ and $p_{2}\left[n\right]$ is illustrated. First, photodetector data are sampled at a rate of $2f_{0}$ samples per second. Then, the decimation block separates the input data into even- and odd-indexed sequences. A low-pass filter is applied to reject electronic noise and frequencies above 3 kHz. Next, interpolation is performed using an expander with a factor of $L_{e}=8$. Finally, sample shifting is used to synchronize both sequences. In the same figure (center, in blue), the PI (proportional-integral) controller is depicted, similar to the one used by [19]. Additionally, the VS/SM observer, which demodulates θ(t), is highlighted inside the magenta rectangle. The PI and VS/SM controllers will be presented in the following sections.

### 2.2. Obtaining the Controlled Signal and PI Controller

Essentially, the purpose of the OVS described in Figure 1 is to measure the voltage $V_{S}\left(t\right)$ by using the nonlinear observer circuit shown in Figure 5, which is implemented by software. As will be seen later, this observer works with two quadrature phase interferometry inputs [46]. Although it is not a necessary condition for this tool, if the quadrature inputs are already stabilized against external environmental disturbances, the observer can operate with greater detection speed, in real time, since tasks such as quadrature correction performed digitally are time consuming. Actually, $V_{\pi S}$ can vary with temperature, causing inaccuracies in the measurement procedure. In addition, the method described in Section 2.1 assumes that (10) is satisfied (i.e., $V_{m}=V_{\pi m}/4$), but in practice, $V_{\pi m}$ varies with temperature (mainly due to the presence of natural birefringence in $V_{\pi m}$ of the OPM [60]). Additionally, the adequate operation of the optical sensors depends on the synchronous acquisition process, which must be precisely tuned to sample v(t) at the instants of maximum and minimum of $V_{m}\left(t\right)$. A simple PI feedback control system can be used to compensate for variations in $V_{\pi S}$ and $V_{\pi m}$, and also compensate for timing inaccuracies in the synchronous acquisition.

Optical phase shift in (10) can be rewritten to include a disturbance Δv as:

(18) $$\psi \left(t\right)=\frac{\pi }{V_{\pi m}}\left(\frac{V_{\pi m}}{4}+\Delta v\right)\cos\left(2\pi f_{0}t\right).$$

The term $V_{\pi m}/4+\Delta v$ in (18) represents the voltage applied to the electrodes of OPM. The control should reduce Δv to zero.

The amplitude of ψ(t) is then given by:

(19) $$\frac{\pi }{4}+\frac{\pi \Delta v}{V_{\pi m}}=\frac{\pi }{4}+\frac{\delta }{2},$$

where $\delta /2=\pi \Delta v/V_{\pi m}$ (in radians). In this case, normalized signals in (17) can be expressed using these new variables as:

(20) $$\begin{array}{cc} p_{1}\left[n\right] & =-\cos\left(\theta \left(nT_{s}^{\prime }\right)+\frac{\delta }{2}\right)\\ p_{2}\left[n\right] & =-\sin\left(\theta \left(nT_{s}^{\prime }\right)-\frac{\delta }{2}\right). \end{array}$$

The Lissajous curve obtain from (20) is an ellipse equated for:

(21) $$p_{1}^{2}\left[n\right]+p_{2}^{2}\left[n\right]+2p_{1}\left[n\right]p_{2}\left[n\right]\sin\left(\delta \right)=\cos^{2}\left(\delta \right),$$

where if δ/2=0, the circumference equation appears.

**Figure 6:**
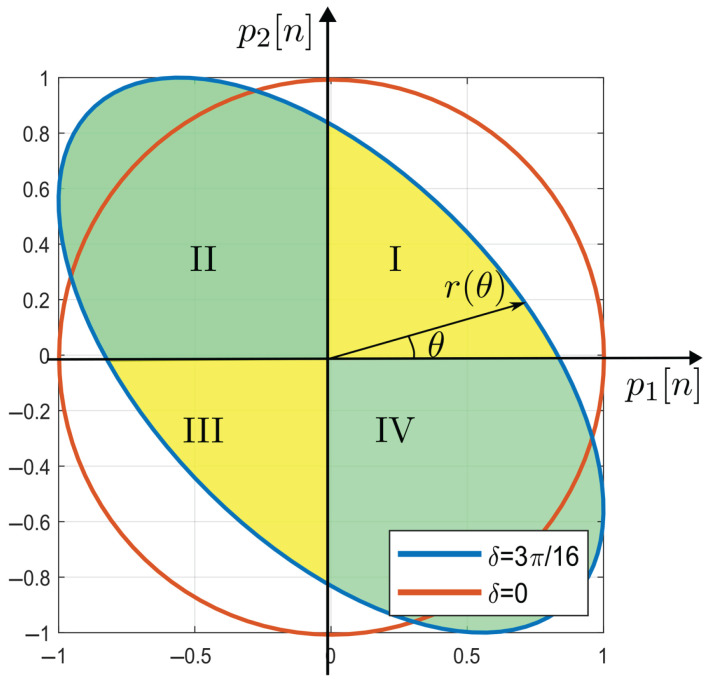
Lissajous curve for different δ.

Figure 6 shows an orange line circumference, obtained when δ=0, and a blue-colored line ellipse, when δ≠0. The yellow- and green-colored areas inside the ellipse for each quadrant are defined as I, II, III, and IV. Naturally, it is possible to assert that: (a) the sum of II and IV is greater than the sum of I and III when δ≠0, and (b) II + IV = I + III when δ=0. Based on this concept, a new control variable

(22) W=(II+IV)−(I+III)

is defined, which must reach a set point of zero.

Defining r(θ) as the distance from the origin to any point on the ellipse, the following equation can be written:

(23) $$r^{2}\left(\theta \right)=p_{1}^{2}\left[n\right]+p_{2}^{2}\left[n\right].$$

Then, substituting (20) in (23), and using trigonometric identities, the next expression can be shown:

(24) $$r^{2}\left(\theta \right)=1-\sin\left(2\theta \right)\sin\left(\delta \right).$$

Areas I, II, III, and IV can be calculated using a polar coordinate system with respect to θ. Integral calculus is used to obtain the areas:

(25) $$Area=\;\int \int \;r\left(\theta \right)\;d\theta \;dr=\frac{1}{2}\int r^{2}\left(\theta \right)\;d\theta .$$

Substituting (24) into (25) and solving for each quadrant, the following areas are obtained:

(26) $$\begin{array}{cc} I & =\frac{1}{2}\int_{0}^{\frac{\pi }{2}}r^{2}\left(\theta \right)\;d\theta =\frac{\pi }{4}-\frac{\sin(\delta )}{2}\\ \mathrm{II} & =\frac{1}{2}\int_{\frac{\pi }{2}}^{\pi }r^{2}\left(\theta \right)\;d\theta =\frac{\pi }{4}+\frac{\sin(\delta )}{2}\\ \mathrm{III} & =\frac{1}{2}\int_{\pi }^{\frac{3\pi }{2}}r^{2}\left(\theta \right)\;d\theta =\frac{\pi }{4}-\frac{\sin(\delta )}{2}\\ \mathrm{IV} & =\frac{1}{2}\int_{\frac{3\pi }{2}}^{2\pi }r^{2}\left(\theta \right)\;d\theta =\frac{\pi }{4}+\frac{\sin(\delta )}{2}. \end{array}$$

With that, (22) can be written as:

(27) W(δ)=2sin(δ).

In practice, areas are calculated using summation in the processing unit myRIO-1900. Area I, for example, is calculated as follows:

(28) $$I=\frac{1}{2}\int_{0}^{\frac{\pi }{2}}r^{2}\left(\theta \right)\;d\theta =\frac{1}{2}\lim_{n\to \infty }\sum_{i=1}^{n}\Delta \theta r^{2}\left(\theta_{i}\right),$$

where Δθ=(π/2−0)/n. The result in (28) is better when the sampling frequency is greater. Then, I is calculated for a sufficiently large n=N as:

(29) $$\begin{array}{cc} I & =\frac{\pi }{4}\left(\frac{r^{2}\left(\theta_{1}\right)+r^{2}\left(\theta_{2}\right)+\cdots +r^{2}\left(\theta_{N}\right)}{N}\right)\\ I & =\frac{\pi }{4}\mu \left(r^{2}\left(\theta_{i}\right)\right), \end{array}$$

where μ is the arithmetic mean. In the same way, other areas can be calculated.

For the particular case when δ≈0, W(δ) exhibits an approximate linear dependence with δ as follows:

(30) W(δ)=2δ.

From Equation (19), *W* can be rewritten as:

(31) $$W\left(\Delta v\right)=\frac{4\pi \Delta v}{V_{\pi m}}.$$

**Figure 7:**
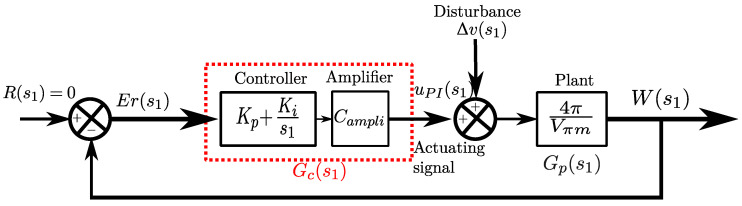
PI control block diagram.

Note that if there is any disturbance, Δv≠0, and the quadrature condition is not reached. Therefore, Figure 7 presents the control system with reference equal to zero and compensation for disturbances, where $s_{1}$ represents the Laplace variable. The controlled signal $u_{PI}$ compensates for Δv to maintain *W* at zero.

In practice, the PI controller is discretized, where the integral part is calculated using the trapezoidal method and the difference equation is then implemented in myRIO. Finally, parameters $K_{p}=0.01$ and $K_{i}=0.1$ are manually tuned. The amplifier after the controller multiplies the controller output by a factor of $C_{ampli}=4.35$.

**Figure 8:**
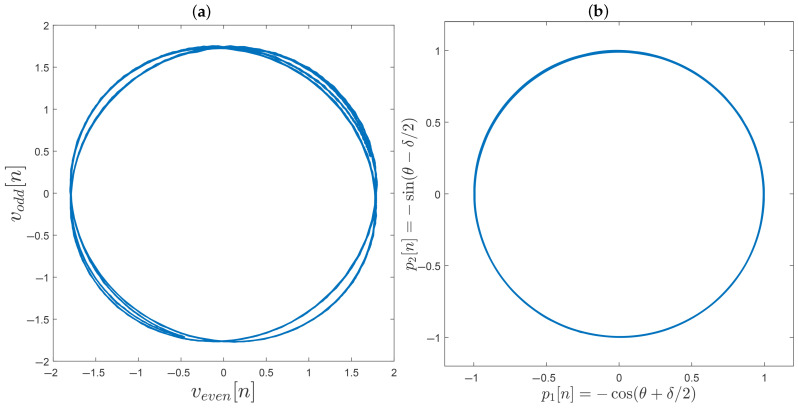
Obtaining the strictly phase quadrature condition: (**a**) Lissajous curve of odd and even signals; (**b**) Lissajous curve of the odd and even signals after filtering, synchronization, interpolation, and control application.

Figure 8a shows a Lissajous curve obtained after the decimation of v(t). On the right, in Figure 8b, a Lissajous curve generated by $p_{1}\left[n\right]$ and $p_{2}\left[n\right]$ is shown after filtering, synchronizing, normalizing, interpolating, and control applying the even and odd sequences from v(t). Thus, the quadrature signals are corrected to become a pair of strictly quadrature signals, as in (17).

The results demonstrate a robust response in the laboratory environment where the interferometer was mounted, maintaining signal quadrature while demodulated voltage measurements were obtained. The model was successfully tested for small variations of error parameter (δ≈0); however, employing a nonlinear control approach (instead of linearizing W=2sin(δ) in (27)) could be an interesting improvement.

This work proposes using a sliding mode observer, which includes nonlinear controller, to demodulate θ(t), presenting for the first time the application of this detection method for optical high-voltage sensing. The theoretical formulation is clearly explained in the following subsection.

### 2.3. Sliding Mode Controller

As described in [46], the closed-loop control operating under the so called high-gain approach (HGA) compensates for both the low frequency spurious disturbances and the signal of interest, i.e., it compensates ($\Psi \left(t\right)+\psi_{0}+\vartheta \left(t\right)+\pi /4$), where the information about the interested signal is contained in Ψ(t).

As mentioned in classical theory for SMC [66], a generic dynamical system can be represented by the state space as:

(32) $$\begin{array}{cc} \dot{x} & =h(x,u)\\ u & =\left\{\begin{array}{cc} u^{+}\left(x\right) & \mathrm{If}\;s(x)>0\\ u^{-}\left(x\right) & \mathrm{If}\;s(x)<0, \end{array}\right. \end{array}$$

where $x\in R^{n}$, $h\in R^{n}$, *u* is a scalar control action with a discontinuous function, and s(x) is the sliding variable. The sliding surface is a closed space in the state space defined as {x(t)|s(x)=0}. As explained by [67], the key is to choose a well-behaved function s(x), and then select the feedback control action *u* in such that $s^{2}$ remains a Lyapunov-like function of the closed-loop system.

Signals in (17) feed the θ(t) observer, which consists of the closed-loop VS/SM control system that generates the total phase shift in closed-loop form as follows:

(33) $$\theta \left(t\right)+\Omega_{c}=\Psi \left(t\right)+\psi_{0}+\vartheta \left(t\right)+\frac{\pi }{4}+\Omega_{c},$$

where $\Omega_{c}$ is the compensation phase shift. It follows because from Figure 5 and (17), we can note that variables *g* and $x_{s}$ can be written as:

(34) $$\begin{array}{cc} g=p_{2}\cos\left(\Omega_{c}\right)+p_{1}\sin\left(\Omega_{c}\right) & =-\sin(\theta +\Omega_{c})\\ x_{s}=-p_{1}\cos\left(\Omega_{c}\right)+p_{2}\sin\left(\Omega_{c}\right) & =\cos(\theta +\Omega_{c}), \end{array}$$

where $p_{1}$ and $p_{2}$ represent the continuous-time-domain versions of $p_{1}\left[n\right]$ and $p_{2}\left[n\right]$ in (17). Thus, the goal of the nonlinear control system is to determine the value of $\Omega_{c}$ to keep the virtual system at a stable quadrature phase point such that $\cos(\theta +\Omega_{c})=0$. In this situation, θ(t) is the signal to be compensated by the signal $\Omega_{c}$.

The sliding surface is selected based on the previously obtained $x_{s}$, as follows:

(35) $$x_{s}\left(t\right)=s\left(x\right)=\cos\left(\theta +\Omega_{c}\right).$$

Computing the time-derivative of s(x)

(36) $${\dot{x}}_{s}=\dot{s}=-\left(\dot{\theta }+{\dot{\Omega }}_{c}\right)\sin\left(\theta +\Omega_{c}\right),$$

and by substituting *g* from (34) in (36), it becomes

(37) $${\dot{x}}_{s}=\dot{s}=g\dot{\theta }+g{\dot{\Omega }}_{c}.$$

In this work, the selected control law to ensure that the system trajectories (state variables) reach the sliding surface in a finite time and remain on it permanently is:

(38) $$u=-Dsgn(x_{s}g),$$

where *u* is a scalar control action with discontinuous function (as mentioned before), *D* is a strictly positive constant representing the overall closed-loop gain, and sgn() is the sign function. Considering the control action $u\left(t\right)={\dot{\Omega }}_{c}$ (shown in Figure 5), a sufficient condition to guarantee the reachability criterion, ensuring that the system converges to the sliding surface s(x)=0 for any g≠0, is $D>|\dot{\theta }\left(t\right)|$, as discussed in [43]. By satisfying the reachability criterion $d\left(s^{2}\left(x\right)\right)/dt=s\left(x\right)\dot{s}\left(x\right)\leq -\eta \left|s\left(x\right)\right|$ (η is positive constant), the surface becomes an invariant set, as described in [67].

The Lyapunov stability can be used to demonstrate points where s=0 are asymptotically stable [26]. Let us choose scalar candidate function $H\left(s\right)=s^{2}/2$ with equilibrium points in s=0. Due to the periodicity of the sliding variable, it has multiple zeros, which can be expressed as

(39) $$\theta +\Omega_{c}=\left(2k+1\right)\pi /2,$$

where k∈Z. The candidate function is defined over an open ball of radius π/2 centered in (39) [26]. Thus, a general open ball, for an arbitrary point given in (39), can be written as:

(40) $$B_{R_{k}}=\left\{\theta +\Omega_{c}|k\pi <\theta +\Omega_{c}<k\pi +\pi \right\}.$$

According to [67], the equilibrium points under analysis are stable if two criteria are met. Since H(s)>0 and 0 in (39), for each ball, H(s) is positive definite, satisfying the first condition.

To meet the second condition, the time derivative of H(s) is analyzed. Multiplying (35) by (36), and substituting (34), the following expression is obtained:

(41) $$\dot{H}\left(s\right)=s\dot{s}=\left(\dot{\theta }+{\dot{\Omega }}_{c}\right)sg.$$

Substituting $u={\dot{\Omega }}_{c}=-Dsgn\left(sg\right)$ from (38) in (41), we obtain

(42) $$\dot{H}\left(s\right)=s\dot{s}=\dot{\theta }sg-Dsgn\left(sg\right)sg.$$

Using identities for sign and absolute functions, the expression (42) becomes

(43) $$\dot{H}\left(s\right)=s\dot{s}=\left(\dot{\theta }sgn\left(sg\right)-D\right)\left|sg\right|,$$

where, since $\dot{\theta }sgn\left(sg\right)\leq \left|\dot{\theta }\right|$, and both *D* and |sg| are positive, the following inequality can be obtained:

(44) $$s\dot{s}=\left(\dot{\theta }sgn\left(sg\right)-D\right)\leq \left(\right|\dot{\theta }|-D)|sg|.$$

Therefore, if $D>|\dot{\theta }|$, $\eta =-(|\dot{\theta }|-D)|sg|$ is strictly positive, and

(45) $$\dot{H}\left(s\right)=s\dot{s}\leq -\eta \left|s\right|.$$

Thus, the time derivative of H(s) is negative definite for g≠0. Now, note that in all balls defined in (40) for any k∈Z, from the definition of *g* in (34), it follows that the condition g≠0 holds. Consequently, H(s) qualifies as a Lyapunov function, and the equilibrium under analysis in (39) is locally asymptotically stable for all balls. Ensuring that $D>|\dot{\theta }|$ guarantees robustness against disturbances and dynamic uncertainties while maintaining the sliding surface.

In the next section, experimental results will be shown.

## 3. Results

**Figure 9:**
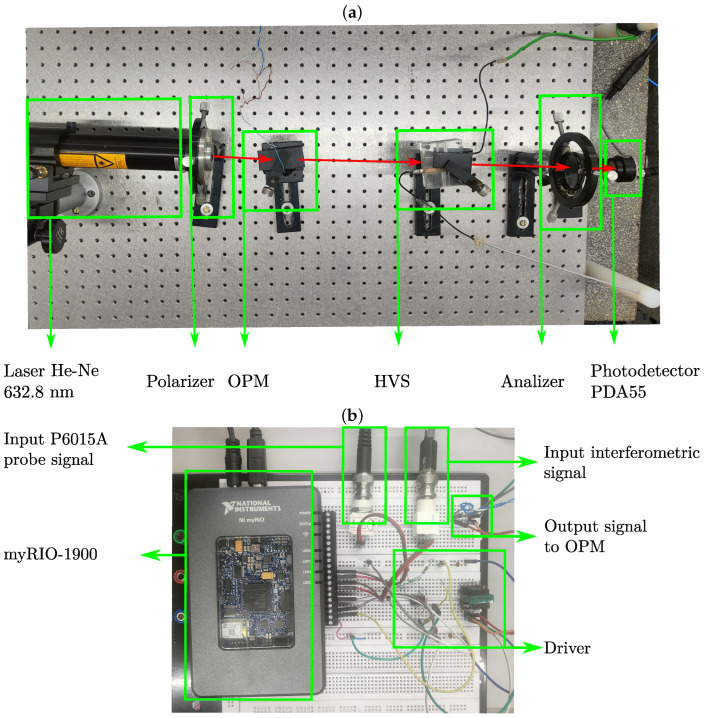
Optical hardware and digital processor of the experimental setup: (**a**) optical elements on breadboard; (**b**) myRIO-1900 and driver signal amplifier.

The optical elements of the experimental setup illustrated in Figure 9a were mounted on a breadboard (Newport-M-SG-23-2). The light source is a 632.8 nm He–Ne laser with an output of 20 mW. The OPM and HVS half-wave theoretical voltage values are $V_{\pi m}$ = 64.92 V and $V_{\pi S}$ = 3.77 kV, respectively. The system was designed to keep the feedback control loop enabled when the measured voltage exceeds the experimental $V_{\pi S}$ value, i.e., when the necessary condition $V_{S}\left(t\right)>V_{\pi S}$ is met.

The output interferometric light is then detected by a photodetector Thorlabs PDA55 (Newton, NJ, USA). The analog signal from the detector is acquired by a myRIO-1900 card, as illustrated in Figure 9b, which has 12 bits accuracy in analog input, and for this paper was set to 30 kHz of sampling frequency. The same figure shows a driver circuit, which amplifies the feedback signal that is applied to the OPM Pockels cell. As can be seen, the architecture of both the optical hardware, the control feedback loop for the OPM modulator, and the signal processing system is extremely simple and inexpensive.

The application is primarily used for voltage measurement and power line harmonic distortion analysis. The effective $V_{S}\left(t\right)$ applied to the HVS was measured by a calibrated high-voltage (HV) probe (Tektronix P6015A, OR, USA) and compared to the demodulated signal produced by our scheme.

### 3.1. Photodetected Signal

The interferometric signal has been detected by an analog input channel, which allows up to ±10 V signal in differential mode. The detected signals were then processed according to the scheme illustrated in Figure 5, which was performed on the LABVIEW program.

The program was set to process 7500 data points at a time. This work used signals $V_{S}\left(t\right)$ with 60 Hz frequency. For 30 kHz sampling frequency, each period is composed of 30,000/60=500 data points. Thus, 7500/500=15 cycles of interferometric signal could be shown for each group of data on a display.

**Figure 10:**
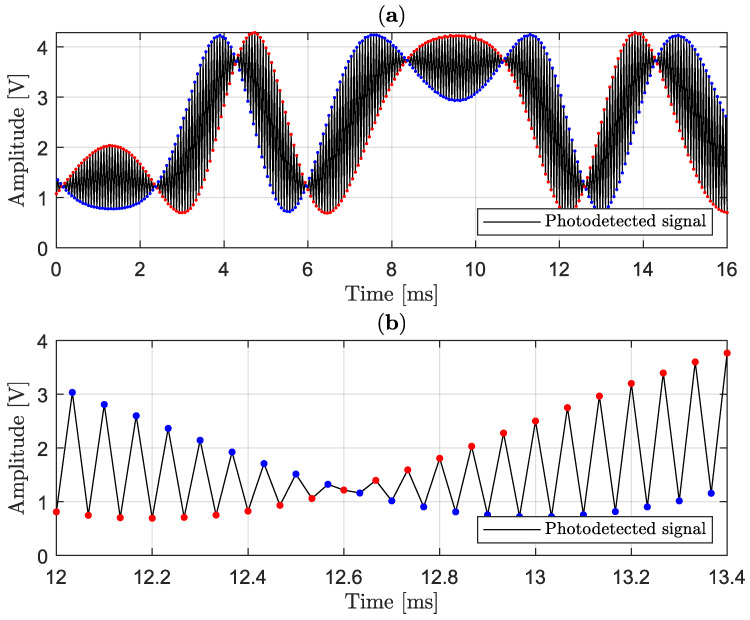
Experimental in-phase and quadrature envelopes of the interferometric signal and the interlaced acquisition: (**a**) photodetected signal sampled at 30 kHz; (**b**) zoom.

Figure 10a shows a typical photodetected signal sampled by the myRIO board and drawn by using the MATLAB R2024b program. In the detail shown in Figure 10b, one cycle of sampled signal can be seen. The red and blue groups of alternating points are shown in order to demonstrate the switching action that must be performed by OPM-fed backed cell (plus interpolation process) in order to generate quadrature signals. In Figure 10b, it can be seen clearly that there is 1/30 ms between two consecutive data points.

### 3.2. Output Signal Using Sign and Sigmoid Functions

As studied in Section 2.3, the reachability criterion is reached when $D>\;|\dot{\theta }|$. Results are analyzed for 60 Hz periodic signals applied in the HVS cell. Remembering

(46) $$\begin{array}{cc} \theta (t) & =\Psi \left(t\right)+\psi_{0}+\vartheta \left(t\right)+\frac{\pi }{4},\\ \theta (t) & =\pi \frac{V_{S}\sin\left(2\pi 60t\right)}{V_{\pi S}}+\psi_{0}+\vartheta \left(t\right)+\frac{\pi }{4}, \end{array}$$

where $\psi_{0}+\vartheta \left(t\right)$, we can assume that both terms are low-frequency signals (<15 Hz). For measured peak voltage $V_{S}$ around one and double of half-wave voltage of HVS, $|\dot{\theta }{|}_{max}$ could be estimated as:

(47) $$|\dot{\theta }{|}_{max}=4\pi^{2}60\approx 2400.$$

For this work, VS/SM gain D=5000 will be chosen. After that, and following the diagram in Figure 5, the signal is then zero-phase filtered, by using a second-order Butterworth bandpass filter with passband between 15 Hz and 3000 Hz. Finally, with the purpose of accurately measuring the value of $V_{S}\left(t\right)$, the filter output is multiplied by $V_{\pi S}/\pi$ factor, where $V_{\pi S}=4.03$ kV was the experimental value rigorously estimated for this HVS cell using the methodology proposed in [18] (presenting a 5.5% discrepancy relative to the $V_{\pi S}=3.77$ kV theoretical value).

In the following items, spectrum graphs are plotted applying discrete Fourier transform (DFT) with $2^{15}$ points and Hanning window, over 10 cycles of signal according to IEC 61000-4-7:2002 [68]. On the other hand, THD calculation was carried out considering the first 50 harmonics recommended by [69].

It is worth remembering that the demodulated signals obtained using the scheme proposed in this work are compared with the original signals measured using as reference a P6015A probe positioned in the HVS cell.

**Figure 11:**
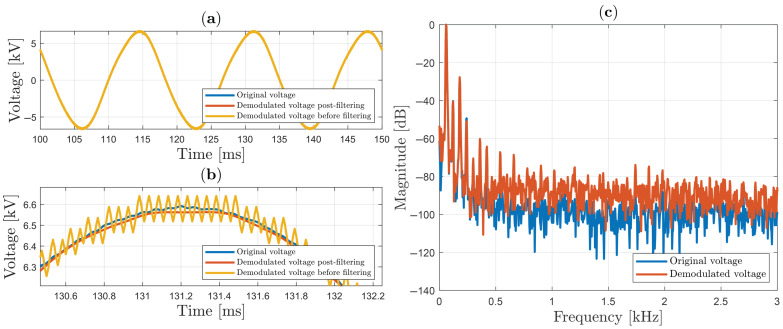
Sinusoidal voltage using sign function in SMC: (**a**) original and demodulated voltage; (**b**) zoom; (**c**) spectrum analysis.

In Figure 11, the sinusoidal signal is shown, with 13.2 kV peak-to-peak (or 6.6 kV peak), which is applied on the HVS cell in the interval between 100 ms and 150 ms. As will be seen, the chattering effect [46] is present in the yellow signal, as a small oscillation generated when the “sign” function is employed as a switch in SMC. These oscillations can be reduced by using an additional bandpass filter. Thus, in Figure 11a, three curves are illustrated: the original signal (in blue color), the demodulated signal with post-filtering (in red color), and the demodulated signal before filtering (in yellow color). The demodulated signal before filtering and demodulated post-filtered signal are both multiplied by $V_{\pi S}/\pi$ factor. When the three signals are plotted on the same scale, the curves overlap due to the great agreement between the results (making the differences between them imperceptible).

A zoom of the three curves (between approximately 130.6 ms and 132.2 ms) is shown in Figure 11b, and helps to understand the differences between the results. In fact, the demodulated signal post-filtering has approximately 13.1 kV peak-to-peak; thus, the relative discrepancy is 0.44%.

The peak-to-peak value is calculated as the difference between the maximum and minimum values of the voltage waveform. In this case—and similarly for the THD relative discrepancy—10 cycles (from the 15 original processed cycles) were analyzed in each iteration to comply with IEC 61000-4-7:2002 [68]. Thus, peak-to-peak relative discrepancy was estimated as

(48) $$\frac{|{\mathrm{PTP}}_{\mathrm{probe}}-{\mathrm{PTP}}_{\mathrm{OVS}}|}{{\mathrm{PTP}}_{\mathrm{probe}}}\times 100,$$

where PTP stands for peak-to-peak. Thus, since ${\mathrm{PTP}}_{\mathrm{probe}}$ is always positive, the discrepancy will also be positive.

The spectra of the signal and the demodulated signal with post-filtering are shown in Figure 11c, between 0 and 3 kHz, and will be used in the next items for the THD analysis. This result shows that the sinusoidal signal generated at the output of the high-voltage transformer presents some deformation; in particular, it contains significant spectral lines above −40 dB at 60 Hz (obviously), 120 Hz, and 180 Hz.

**Figure 12:**
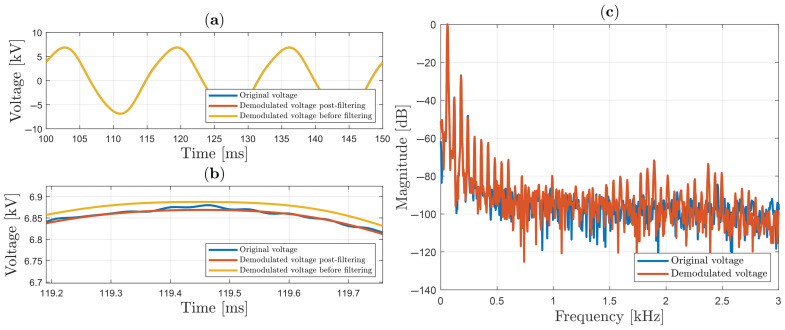
Sinusoidal voltage using sigmoid function (ϵ=0.065) in SMC: (**a**) original and demodulated voltage; (**b**) zoom; (**c**) spectrum analysis.

In Figure 12, a 13.7 kV peak-to-peak sinusoidal waveform is applied in the HVS cell, but in this case, a “sign” function in SMC is replaced by sigmoid (“sgm”) function:

(49) $$sgm\left(*\right)=\frac{*}{|*|+\epsilon },$$

where ϵ is a constant value very close to zero and strictly positive. The use of the “sgm” function is an almost mandatory procedure in practical experiments involving SMC in order to reduce the chattering effect. Results corresponding to those in Figure 11 are shown in Figure 12a–c. As can be seen, using ϵ=0.065, oscillation because of chattering is reduced before filtering out the signal. In this situation, the peak-to-peak discrepancy is only 0.065%.

Although the results obtained with the sigmoid function outperform those achieved with the sign function in terms of peak-to-peak discrepancy, this paper employs the sign function combined with the output bandpass filter for the subsequent measurements.

### 3.3. Measurement of Non-Sinusoidal Periodic Signals

Two non-sinusoidal periodic signals were generated from the distortion caused by the action (low-pass filtering and/or intrinsic nonlinearities) of the dry electromagnetic transformer itself, when applying triangular and square waveforms through its primary winding. These signals will be referred to here simply as “Waveform 1” and “Waveform 2”, respectively. Similarly to the first sinusoidal case, the chattering effect appears as oscillation when the SMC uses the sign function as switch.

**Figure 13:**
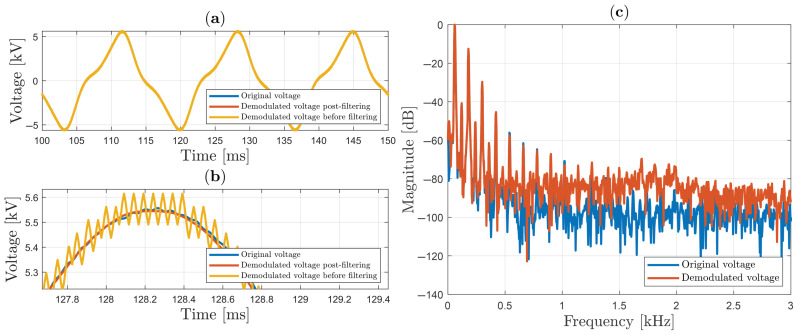
“Waveform 1” voltage using sign function in SMC: (**a**) temporal analysis; (**b**) zoom; (**c**) spectrum analysis.

Figure 13a plots the three signals—i.e., the original, the demodulated before filtering, and the post-filtered demodulated—when a voltage equal to 11.1 kV peak-to-peak of “Waveform 1” is applied to the HVS. The zoom plot shown in Figure 13b shows that there is excellent agreement between results, with relative errors of only 0.12% peak-to-peak between the original signal and post-filtered demodulated signal. The spectral plots shown in Figure 13c reveal that this waveform presents a higher spectral content than the cases in Figure 11 and Figure 12, and that first odd harmonic frequencies are higher in respect to sinusoidal case.

**Figure 14:**
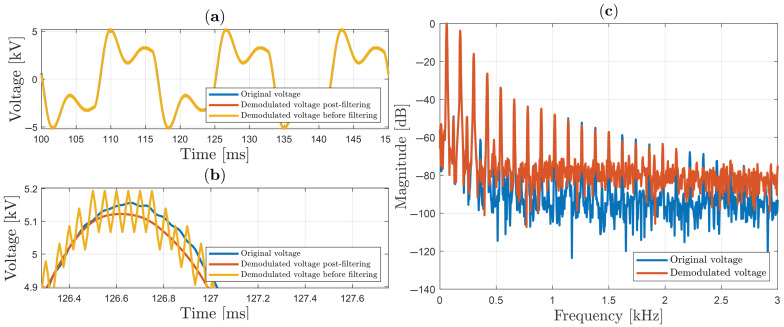
“Waveform 2” voltage using sign function in SMC: (**a**) temporal analysis; (**b**) zoom; (**c**) spectrum analysis.

In the second case, measurements similar to the previous case are presented in Figure 14a, when a voltage equal to 10.3 kV peak-to-peak of “Waveform 2” is applied to the HVS. Even with such distortion (with high THD value, as will be seen later), the zoomed plot shown in Figure 14b shows a small relative error, around 0.43% peak-to-peak. In addition, it can be noted that odd harmonic components of “Waveform 2” predominate throughout the bandwidth of 3 kHz.

### 3.4. OVS Linearity Analysis and THD Analysis

**Figure 15:**
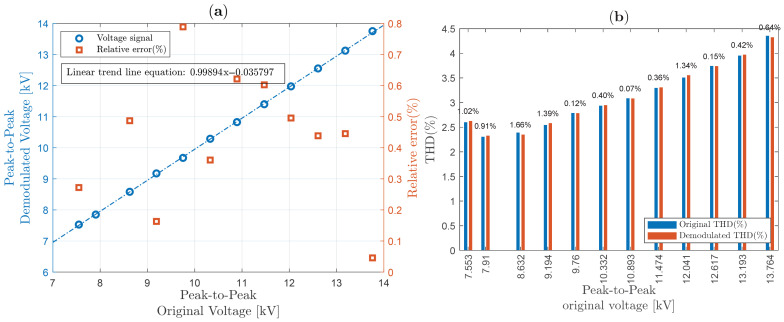
System linearity, THD, and relative error between original and demodulated for a sinusoidal voltage: (**a**) system linearity; (**b**) THD.

**Figure 16:**
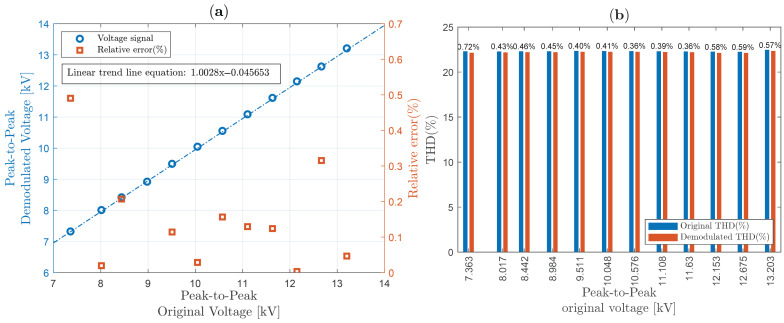
System linearity, THD, and relative error between original and demodulated for a “Waveform 1” voltage: (**a**) system linearity; (**b**) THD.

**Figure 17:**
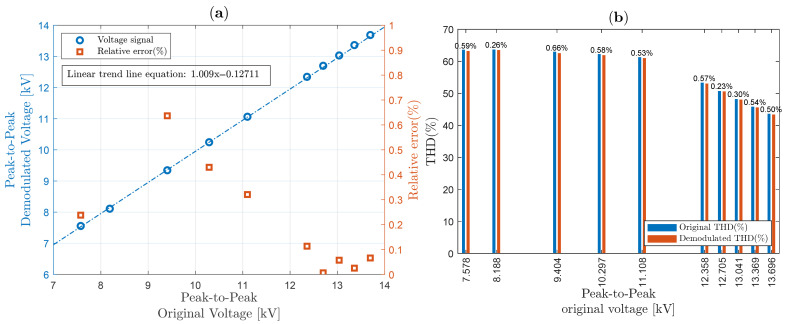
System linearity, THD, and relative error between original and demodulated for a “Waveform 2” voltage: (**a**) system linearity; (**b**) THD.

The IEC 61869-3 standard, relating to HV instrument transformer (including OVSs) power quality measurements, imposes a well-defined accuracy (class dependent) for a voltage between 80% and 120% of its nominal voltage value [50]. Although the electromagnetic transformer used in this work can operate with up to 15 kV RMS, the limit of approximately 7 kV peak was obeyed due to the unavailability of a crystal with larger dimensions, under the risk of dielectric breakdown (through the air, close to the edges of the crystal) due to the high intensity of the applied electric field. Therefore, our OVS system was designed to enable safe operation around 5 kV RMS (±20%), i.e., between approximately 7 kV peak-to-peak and 14 kV peak-to-peak, in order to obtain the linearity graphs shown in Figure 15a, Figure 16a and Figure 17a, for sinusoidal waveforms, “Waveform 1” and “Waveform 2”, respectively.

Figure 15a shows (in red squares) that the relative errors are less than 0.8% for the corresponding voltage range. In addition, with the help of MATLAB, a linear trend was calculated, where the first order factor is close to 1 (with a coefficient of determination, R-squared, equal to 0.99894).

Meanwhile, THD for sinusoidal signal was analyzed and compared in Figure 15b. On top of the bars, relative errors are shown, revealing that in general, relative THD errors are less than 1.7%. Similarly to (48), THD relative discrepancy is computed as

(50) $$\frac{|{\mathrm{THD}}_{\mathrm{probe}}-{\mathrm{THD}}_{\mathrm{OVS}}|}{{\mathrm{THD}}_{\mathrm{probe}}}\times 100,$$

On the other hand, Figure 16a shows the linearity graph when “Waveform 1” is applied to the HVS, showing that the relative errors are less than 0.8%. Recall that “Waveform 1” in the secondary winding of the high-voltage transformer was defined for a triangular waveform applied to the primary winding. The calculated linear trend in this case shows the first-order factor is close to 1. On the right, in Figure 16b, it can be seen that the THD of “Waveform 1” is approximately 22%. Comparing the THD between the original value obtained by the probe and our proposed scheme, the relative errors—shown at the top of the bars—are less than 0.8%.

At last, “Waveform 2” linearity is presented. In Figure 17a, the linearity curve is shown, where the relative errors are less than 0.8%. As in the previous cases, the linear trend has a first-order coefficient close to 1. On the right, Figure 17b, note that THD is decreasing from around 64%, probably due to the nonlinearity of the voltage elevation system. However, the new proposed demodulation scheme tracks this behavior, resulting in relative errors of less than 0.8%.

It is worth mentioning that the linearity measurement was performed by using the sign function as a switch in SMC for all cases. In addition, it must be noted that voltage range is higher than $V_{\pi S}$ (in the condition of high modulation depth). However, if the measured signals $V_{S}\left(t\right)$ were below $V_{\pi S}$ (with low modulation depth), the Lissajous curve shown in Figure 6 would not be closed, leading to incorrect calculations in (26), since a portion of the ellipse is missing. In principle, it is still possible to overcome this situation if the values of bias voltage *M* and visibility *V* in (9), as well as the control signal value applied to the OPM cell when working under normal operation conditions of $V_{S}\left(t\right)>V_{\pi S}$, are all stored in memory. Then, we must turn the PI controller off and use these stored values to measure $V_{S}\left(t\right)<V_{\pi S}$. This solution could be only applied if the interferometer environment is well behaved (mainly in terms of temperature) because, for external spurious and random environmental influences, the stored control signals could not compensate highly varying disturbances.

### 3.5. Sliding Variable Behavior

**Figure 18:**
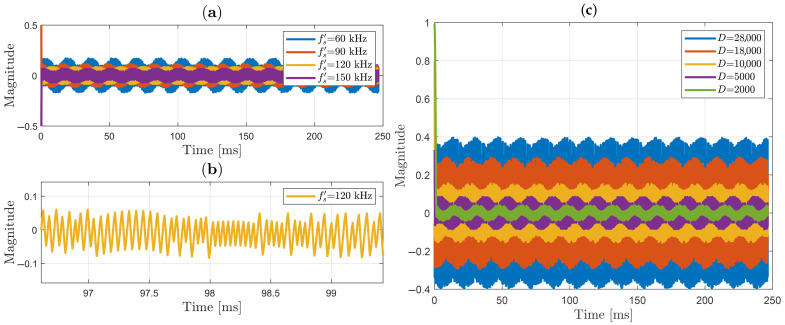
Variation of chattering magnitude with time: (**a**) sliding variable using different sampled frequencies for D=5000; (**b**) zoomed sliding variable sampled at 120 kHz; (**c**) sliding variable using different *D* constants.

In Section 2.3 the sliding variable $s=\cos(\theta +\Omega_{c})$ was defined, where the sliding controller works keeping *s* at zero. Figure 18a shows sliding variable performance when a sign function is used as a switch in the observer. First, note that *s* is not strictly zero (as is clearly shown in Figure 18b) but oscillates around zero due to non-instantaneous and imperfect switching in practice, leading to chattering. It can also be observed that the amplitude of the sliding variable has an inverse relationship with the sampling frequency; that is, its amplitude decreases as the sampling frequency of the signals feeding the observer increases. This behavior can be analyzed by considering that the observer was implemented digitally. Thus, the control signal, according to Equation (38), is expressed as:

(51) $$u=-Dsgn(x_{s}g),$$

where $x_{s}$ was defined in (35). In Figure 5 (in the observer), the control action is $u={\dot{\Omega }}_{c}$. By substituting these expressions in (51), we obtain:

(52) $${\dot{\Omega }}_{c}=-Dsgn\left(sg\right).$$

The observer is fed by the functions in (17), which have a sampling period of $T_{s}^{\prime }$. Thus, expression (52) can be written in its discrete form as:

(53) $$\frac{\Omega_{c}\left[n\right]-\Omega_{c}\left[n-1\right]}{T_{s}^{\prime }}=-Dsgn\left(s\left[n-1\right]g\left[n-1\right]\right).$$

Separating current values from preview values, and using $T_{s}^{\prime }=1/f_{s}^{\prime }$, (53) becomes

(54) $$\Omega_{c}\left[n\right]=\Omega_{c}\left[n-1\right]-\frac{Dsgn(s[n-1]g[n-1])}{f_{s}^{\prime }}.$$

It should be remembered that the sampling frequency can be adjusted by updating the expander $L_{e}$ factor shown in Figure 5 (left, in black). On the other hand, the sliding variable in discrete time can be presented as

(55) $$s\left[n\right]=\cos(\theta \left[n\right]+\Omega_{c}\left[n\right]),$$

and, substituting (54) in (55), we have

(56) $$s\left[n\right]=\cos\left(\theta \left[n\right]+\Omega_{c}\left[n-1\right]-\frac{Dsgn(s[n-1]g[n-1])}{f_{s}^{\prime }}\right).$$

As demonstrated in Section 2.3, the observer has stable equilibrium points at $\theta +\Omega_{c}=\left(2k+1\right)\pi /2$ for k∈Z. Therefore, we can consider that, on the sliding surface, (56) can be written as

(57) $$s\left[n\right]=\cos\left(\frac{(2k+1)\pi }{2}-\frac{Dsgn(s[n-1]g[n-1])}{f_{s}^{\prime }}\right),$$

which, after applying trigonometric identities, can be expressed as

(58) $$s\left[n\right]=\sin\left(\frac{Dsgn(s[n-1]g[n-1])}{f_{s}^{\prime }}\right).$$

In (58), it is shown that if observer operates close to the sliding surface, the amplitude of the sliding variable *s* decreases with higher sampling frequency. Additionally, *s* can take both positive and negative values due to the presence of the sign function. It is worth mentioning that, in practice, $\theta \left[n\right]+\Omega_{c}\left[n-1\right]$ may not be exactly equal to (2k+1)π/2, so a small phase term should be considered in both (58) and (54). These two characteristics give *s* a stochastic behavior.

Considering that from (34), g[n−1]=−1 at the stable equilibrium points at $\theta +\Omega_{c}=\left(2k+1\right)\pi /2$ for k∈Z, for small values of $D/f_{s}^{\prime }$, as $\sin\left(D/f_{s}^{\prime }\right)\approx D/f_{s}^{\prime }$ for $|D/f_{s}^{\prime }|<<1$, note that (58) can be approximately described by:

(59) $$s\left[n\right]=-\frac{D}{f_{s}^{\prime }}sgn\left(s\left[n-1\right]\right).$$

Now, let $H\left[n\right]=s{\left[n\right]}^{2}/2$ (Lyapunov function from Section 2.3) and calculate for s[n−1]≠0:

(60) $$H\left[n\right]-H\left[n-1\right]=\frac{1}{2}{\left(-\frac{D}{f_{s}^{\prime }}\right)}^{2}-\frac{s{\left[n-1\right]}^{2}}{2}.$$

Therefore, H[n]−H[n−1]<0 only if $|s\left[n-1\right]|>D/f_{s}^{\prime }$ and H[n]−H[n−1]=0 for $\left|s\left[n-1\right]\right|=D/f_{s}^{\prime }$. This means that, as $\left|s\left[n-1\right]\right|=D/f_{s}^{\prime }$ is not an equilibrium point of (59), s[n] will be an oscillatory signal with amplitude around $D/f_{s}^{\prime }$, that decreases as the sampling frequency $f_{s}^{\prime }$ increases.

Since the applied voltage is demodulated from $\Omega_{c}$, the characteristics of *s*, such as its chattering amplitude, are reflected in the demodulated voltage waveform. This is illustrated in the waveform before filtering shown in Figure 11b.

The sliding variable was also analyzed for different constant values of gain *D*, from (38). It is worth remembering the condition to guarantee reachability criterion is $D>|\dot{\theta }\left(t\right)|$; however, Figure 18c demonstrates that *D* cannot be excessively large, since this would increase the amplitude of *s*. The gain *D* takes into account the measured signal frequency (60 Hz), as seen in Equation (47), which assumes an ideal sinusoidal waveform. However, in practice, the measured signal often contains multiple harmonics, and thus, *D* should be designed to cover sufficient bandwidth while avoiding distortion in the demodulated signal.

### 3.6. Stability Points

**Figure 19:**
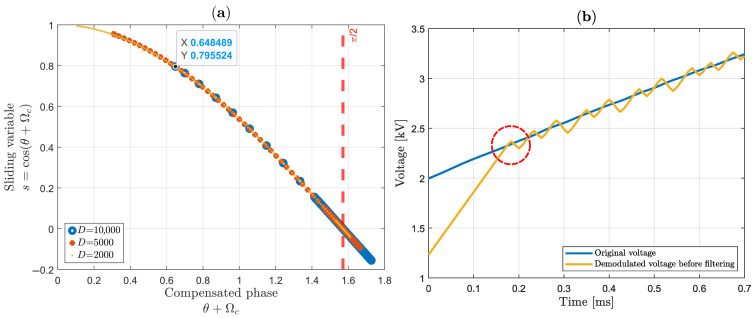
Analysis of establishment time: (**a**) sliding variable for different *D*; (**b**) first instants of demodulated signal using D=5000.

According to [67], if the reachability criterion is satisfied, starting from any initial condition the state trajectory reaches a sliding surface in a finite time. Section 2.3 showed that the system has equilibrium points at $\theta +\Omega_{c}=\left(2k+1\right)\pi /2$; consequently, $s=\cos(\theta +\Omega_{c})$ equals zero at these points. Figure 19a captures one of the instants where *s* reaches the π/2 equilibrium point for three different values of *D* with different starting points. According to [67], the time to reach this sliding surface can be estimated as

(61) $$t_{reach}\leq \frac{\left|s(t=0)\right|}{\eta },$$

where η is a strictly positive number defined in (45). Without loss of generality, we can estimate the time that each curve will take to reach the stability point in $\theta +\Omega_{c}=\pi /2$ (starting from the small pop-up in Figure 19a) by counting the number of points before s approaches zero. Thus, the finite time can be estimated by multiplying this number by a new sampling period ($T_{s}^{\prime }$ in Equation (17)). The results are listed in Table 2.

The finite time formulated in (61) has already predicted these results. Starting from the same point, the curve with higher *D* takes less time to reach the sliding surface. Another important behavior is the amplitude of *s* around the stability point. When using a lower value, D=2000, the sliding variable remains closer to zero, in contrast to D=10,000. This amplitude characteristic is best illustrated in the time domain in Figure 18c. As a summary, a higher *D* can be beneficial for reaching the equilibrium point faster, but it results in higher amplitude of *s* while maintaining the sliding surface. Considering these characteristics and the fact that we are sensing non-ideal signals with the presence of harmonics, D=5000 best satisfies these requirements for this work, as estimated in (47).

Finite time to reach stability can also be seen along the first instants of demodulated signal. Using D=5000, Figure 19b shows that the demodulated signal (yellow line curve) fails to track the original signal (blue line curve) because the system requires approximately 0.2 ms to begin proper tracking. As mentioned in Section 3.1, 15 cycles of 60 Hz (250 ms) measured signal are processing at time, meaning only 100×0.2/250=0.08% of the signal is affected by this establishment time. It is important to comment that the initial point moves in each iteration; consequently, the finite time can be reduced if the initial point is close to any equilibrium point. Furthermore, the system may not remain constrained to the π/2 point indefinitely. Depending on environmental disturbances, the system may leave a stable equilibrium point and then reach another equilibrium point, the choice of which will depend on the initial starting point location.

## 4. Conclusions

In this article, it was demonstrated that a digital sliding mode observer is extremely suitable for application to optical high-voltage measurement in order to demodulate the optical phase shift generated by the optical voltage sensors (OVSs). To the best knowledge of the authors, this is the first time such a strategy has been tested. Since the nonlinear observer requires two input signals in quadrature, a simple PI controller was used both to mitigate the signal fading problem and to generate those signals. The closed-loop OVS system, using the PI controller, was implemented in hardware (which provides a no-time-consuming signal processing, as with its respective digital version), which enabled faster detection of the optical phase shift and provided 3 kHz bandwidth.

As a proof of the observer’s stability, an analysis based on Lyapunov theory was conducted. According to this criterion, the system was shown to be stable at the points (2k+1)π/2 for k∈Z, ensuring the robustness of the observer. The chattering phenomenon associated with sliding mode control was simply addressed by applying a band-pass filter at the observer output. However, this also becomes a limitation in our OVS, as it restricts the sensor’s bandwidth (but, contrary to regular applications in optical interferometry, where bandwidth of dozens of kHz or higher may be used, for power line operation at 60 Hz, this 3 kHz bandwidth may be considered good enough for most requirements). In our case, good results were obtained using a band-pass filter with a high cut-off frequency at 3 kHz (covering the first 50 harmonics of a 60 Hz signal), for voltages with up to 65% THD. Nevertheless, the sensor may not perform well for measuring fast and high amplitude transients (such as extremely short time voltage surges—on the order of 40 µs pulse widths), as it would lack sufficient bandwidth for such signals, an important characteristic desired in the area of power quality (for example).

Additionally, an analysis of the relationship between chattering amplitude, observer gain *D*, and the sampling frequency of the input signals was conducted. It was shown that the gain D must be greater than the time derivative of the phase to be compensated within the observer; however, this constant cannot be excessively large, as it would increase chattering amplitude. On the other hand, higher sampling frequencies reduce chattering amplitude, which is beneficial.

Moreover, in the study of sliding mode systems, it is important to consider the time it takes for the controller to bring the state variables to the sliding surface. Based on several experimental tests and mathematical analysis, it was found that good performance can be achieved by using a gain of D=5000 and a sampling frequency of $f_{s}^{\prime }=120$ kHz.

Due to the need for a band-pass filter to reduce chattering in the demodulated voltage signal, it is reasonable to direct future observer optimizations toward obtaining a chattering-free demodulated signal, thereby eliminating the need for the filter. The literature discusses several possible solutions, such as avoiding the use of a discontinuous function in the controller and instead employing smooth functions (e.g., sigmoid or saturation functions). This approach involves replacing the sign function and redoing the Lyapunov stability analysis, as well as the gain and sampling frequency analysis, using the newly selected function to reduce or eliminate chattering. For now, the initial version presented in this paper adopted the basic controller structure based on the sign function.

The demodulated voltages using this new scheme were analyzed and compared with a P6015A high-voltage commercial probe connected to the HVS Pockels cell. The OVS was assembled and tested with 60 Hz voltage signals (sinusoidal or non-sinusoidal periodic voltages), ranging from 7 kV to 14 kV peak-to-peak and with THD below 65%, showing relative errors lower than 0.8% and 1.5%, respectively. The new OVS can be classified as Class 1.0 according to the UNE-EN 60044:7 standard (adapted from the former IEC 60044-7:1999 standard), with voltage errors within ±1%. Finally, it can be concluded that the proposed OVS strategy has great potential for real-time energy quality measurement and monitoring in power systems (particularly for the 13.8 kV class, because the OVSs could be connected directly to the high-voltage transmission line without the use of resistive or capacitive voltage dividers).

## Figures and Tables

**Table 1:** Quantitative comparison with FBG-PZT sensors.

Article/Year	Anti-Environmental Interference	Technology	Peak-to-Peak Relative Error	Bandwidth
[11]/2019	Thermo-optic effect and thermal expansion	FBG-PZT	0.2% (for signals with THD < 10%)	-
[10]/2019	Thermo-optic effect and thermal expansion	FBG-PZT	3%	-
[5]/2021	Thermo-optic effect and thermal expansion	FBG-PZT	-	2.5 kHz
This paper/2025	General environmental perturbation	LN bulk crystal	0.8% (for signals with THD < 65%)	3 kHz

**Table 2:** Estimated time to reach the sliding surface for different *D*.

*D*	Number of Points	Time [ms]
2000	80	0.66
5000	28	0.23
10,000	9	0.075

## Data Availability

The original contributions presented in the study are included in the article. Further inquiries can be directed to the corresponding author.

## Abbreviations

The following abbreviations are used in this manuscript:

OVS	Optical voltage sensor
PI	Proportional-integral
THD	Total harmonic distortion
PT	Potential transformers
FBG-PZT	Fiber Bragg grating—lead titanate zirconate
I/O	Input-output
PID	Proportional-integral-derivative
LMI	Linear matrix inequality
IO	Integrated optical
VS/SM	Variable structure and sliding modes
TS	Takagi–Sugeno
HGA	High gain approach
SMC	Sliding mode control
IEC	International Electrotechnical Commision
EN	European Norm
OPM	Optical phase modulator
HVS	High-voltage sensor
DFT	Discrete Fourier transform

## Appendix A. Mean Blocks

In Figure 5, Mean$\left(*\right)$ blocks appear to obtain DC term *M* and MV products. Since this paper was designed for the $V_{S}\left(t\right)>V_{\pi S}$ condition, the cosine term can take values from −1 to 1 (its complete range). Processing many cycle at times (15 for this paper), possibly to estimate maximum and minimum values for $v_{pd}\left(t\right)$ in each iteration. The maximum value is calculated as $v_{pd}{\left(t\right)}_{max}=M\left(1+V\right)$, while the minimum value is computed as $v_{pd}{\left(t\right)}_{min}=M\left(1-V\right)$. Then, *M* can be calculated as:

(A1) $$M=\frac{v_{pd}{\left(t\right)}_{max}+v_{pd}{\left(t\right)}_{min}}{2}.$$

The *M* value is calculated as the arithmetic mean of the maximum and minimum value of $v_{pd}\left(t\right)$ and is always updating in each iteration, assuring that any variation in *M* cannot affect post-calculations. This constant update over time allows the system to self-calibrate in response to variations in laser output intensity, which would be reflected by a change in *M*.

Using the same condition, even and odd sequences in (16) can be normalized, finding MV. The same as before, estimating the arithmetic mean of the minimum and maximum for each sequence and dividing by itself, the expression (17) can be written [70].
